# Design and initial implementation of the WHO FP umbrella project- to strengthen contraceptive services in the sub Saharan Africa

**DOI:** 10.1186/s12978-017-0335-0

**Published:** 2017-06-15

**Authors:** Rita Kabra, Moazzam Ali, James Kiarie

**Affiliations:** 1 Department of Reproductive Health and Research including UNDP/UNFPA/UNICEF/WHO/World Bank Special Programme of Research, Development and Research Training in Human Reproduction, World Health Organisation, Avenue Appia 20, 1211 Geneva, Switzerland; 2 Department of Reproductive Health and Research, World Health Organisation, Geneva, Switzerland; 3 22 chemin Henry Wissner, 1212, Grand Lancy, Geneva, Switzerland

**Keywords:** WHO family planning guidelines, Contraception, Unmet need, Implementation, Adaptation, Partnerships, Health systems, Capacity building, Sub-Saharan Africa, Sustainability

## Abstract

**Background:**

Strengthening contraceptive services in sub Saharan Africa is critical to achieve the FP 2020 goal of enabling 120 million more women and girls to access and use contraceptives by 2020 and the Sustainable Development Goals (SDG) targets of universal access to sexual and reproductive health (SRH) services including family planning by 2030.

**Method:**

The World Health Organization (WHO) and partners have designed a multifaceted project to strengthen health systems to reduce the unmet need of contraceptive and family planning services in sub Saharan Africa. The plan leverages global, regional and national partnerships to facilitate and increase the use of evidence based WHO guidelines with a specific focus on postpartum family planning. The four key approaches undertaken are i) making WHO Guidelines adaptable & appropriate for country use ii) building capacity of WHO regional/country staff iii) providing technical support to countries and iv) strengthening partnerships for introduction and implementation of WHO guidelines. This paper describes the project design and elaborates the multifaceted approaches required in initial implementation to strengthen contraceptive services.

**Conclusion:**

The initial results from this project reflect that simultaneous application these approaches may strengthen contraceptive services in Sub Saharan Africa and ensure sustainability of the efforts. The lessons learned may be used to scale up and expand services in other countries.

## Background

Unintended pregnancy resulting from the unmet need for family planning/contraception threatens the lives and well-being of women and girls and their families globally [1]. About 225 million women in low and middle-income countries have an unmet need for a modern method of family planning with the highest unmet need being in Sub-Saharan Africa [1–3]. Discounting the partial protection from lactational amenorrhea, unmet need for family planning is particularly high among postpartum women [4] resulting in high levels of unintended pregnancies (estimated 73 million in 2012 [5]) especially in low and middle income countries.

Estimates by Guttmacher Institute indicate that, if all women who want to avoid a pregnancy used modern contraceptives and all pregnant women and their new-borns received care at the standards recommended by WHO the unintended pregnancies would drop by 70%, maternal deaths by 67%, and newborn deaths would decline by 77% (1) 1 as compared to the current situation. Post-partum family planning (PPFP) programs could effectively contribute to achieving these results. Postpartum programs are premised on the assumption that demand for pregnancy prevention is significant following childbirth and that provision of services before discharge or at postnatal visits is a cost-effective solution and critical to achieving results. Studies show that where people feel they are receiving good-quality care, contraceptive use is higher, and that achieving higher standards of quality improves the effectiveness of sexual and reproductive health services and attracts people to use them [6].

To address the low use of contraceptives particularly during the post-partum period and to ensure that decisions about family planning services are informed by the best evidence and field tested practices, World Health Organisation (WHO) has embarked on the “*FP-Umbrella project*”. The project was designed and initiated in November 2015. The overarching goal of the project is to strengthen policy and health systems response to reduce unmet need for contraceptives with special focus on PPFP. The four key approaches for implementation of this project are:

1. Making WHO Guidelines adaptable & appropriate for country use
2. Building capacity of WHO regional/country staff to support implementation of WHO guidelines
3. Providing technical support to countries for adaptation, adoption and use of WHO guidelines
4. Strengthening partnerships for introduction and implementation of WHO guidelines

**Fig. 1:**
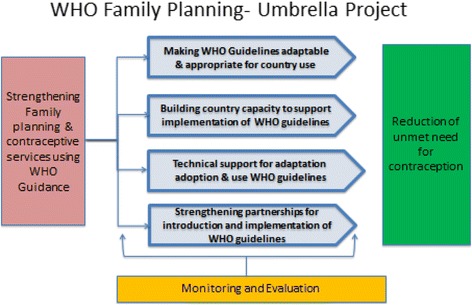
Multi-dimensional approach to strengthen family planning

These approaches are interrelated and designed to address critical gaps in the health system to reduce unmet need for contraception. This paper provides the rationale for this approach and describes initial implementation activities in ten focus countries in Africa.^1^ These ten countries are selected based on the disease burden (unmet need in family planning), political willingness, and commitment at national levels to FP 2020 Goals. Lessons learnt from these countries can be used to scale up and expand practices in other countries Fig. 1.

## Approach 1-Making WHO guidelines adaptable & appropriate for country use

WHO is the lead norms setting agency on health in the United Nations (UN) system. The department of Reproductive Health and Research (RHR) in WHO has developed over 30 guidelines and tools for family planning managers and providers to promote evidence based FP services globally. These include the four cornerstones of family planning guidelines [7]: Medical Eligibility Criteria for contraceptive use (MEC); Selected Practice Recommendations for contraceptive use (SPR) [8]; Decision making tool for Family planning Clients (DMT) [9] and Family planning: A Global Handbook for Providers [10].

Review of lessons learnt from previous guideline introduction activities in countries and discussions with key stakeholders (implementing partners, ministry of health, health care providers, trainers) revealed that the common challenges/barriers that limit the use of WHO FP guidance were lack of guidance for implementation [11] (clarity on what to use due to multiple guidelines, difficulties in interpreting some MEC categories, incorrect interpretation of recommendations), information on what is needed to contextualise or adapt a guideline and lack of documentation of effective implementation approaches.

To respond to these challenges and requests by countries to simplify and promote the use of the four cornerstones of family planning, emphasising quality and human rights principles, WHO RHR has developed simplified derivative tools to enhance implementation of evidence based recommendations in countries. These includes the i) WHO Postpartum Family Planning (PPFP) Compendium [12] that integrates core WHO guidance on safe provision of PPFP methods based on individuals’ needs and context, ii) A simple user friendly Check List [13] to prompt health workers to adhere and implement the principles of quality of care in contraceptive services and information from a human rights perspective and iii) A documentation guide [14] to support program managers and health care providers to identify the success and the impact of the programme and practices and document best practices during adaptation, implementation and scaling up of programmes.

Pilot test results have suggested these new simplified tools and guidelines are user friendly, effective and easy to use. Studies from developing countries confirm that simplified tools that facilitate the application of knowledge at the point of care and thereby simplify adherence to guidelines, can improve practitioner’s performance, and ultimately better patient care [15]. We look forward to countries using these tools to support implementation of WHO FP guidance in 2017.

## Approach 2- Regional capacity building to support implementation of WHO guidance

Despite large-scale initiatives like the Strategic Partnership Programme (SPP) [16] by WHO and UNFPA, to support countries in developing knowledge, tools and national guidelines, the uptake in practice and in improving quality of services at the national level has been limited and not sustained. Limited resources (human and financial) at WHO country offices to support the family planning programmes including implementation of evidence based WHO FP guidelines have been cited as a key challenge for the limited impact.

Under this project, special efforts are directed to build capacity at the regional level with the expectation that this will be cascaded down to national and subnational level by local partners. In this context a regional consultation was organised in Harare, Zimbabwe in July 2016 which was attended by representatives of ministries of health, development partners, civil society, non-governmental organisations, academia and WHO country staff from 18 African countries. Participants were oriented on the latest FP recommendations and guidelines. The teams jointly prepared action plans to adopt and implement the WHO guidance in their countries. In addition, three WHO inter-regional support teams based in Harare, Libreville and Ouagadougou, were identified to support country offices in their efforts to work with ministries of health in updating FP norms and standards.

Considering that information and local expertise is most likely to influence policy makers [17], WHO initiated a program to strengthen technical capacity of the obstetricians and gynaecologists to support the ministry of health (MOH) in interpreting and advocating the use of evidence based FP guidelines. In July 2016, WHO in collaboration with International Federation of Gynaecology and Obstetrics (FIGO) organized a Training of Trainers (ToT) workshop at Johannesburg, South Africa, to orient obstetrics and gynaecologists (approx. 20 participants) on the latest FP recommendations in accordance with the WHO’s MEC 5th edition on the safety and efficacy of contraception. A critical dimension of this training was to create capacity for organizational development so that the trainees can cascade training and facilitate and support ministries of health in adopting WHO evidence based guidelines into national programs. Following this, trainees from Nigeria and Rwanda conducted workshops in their countries to orient their peers on MEC Guidance.

## Approach 3: Technical support for adaptation, adoption and use WHO guidelines

**Fig. 2:**
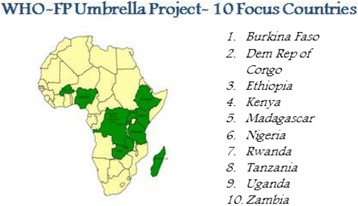
WHO FP-10 focus countries in Africa

Countries develop national standards and practice guidelines by adapting WHO guidelines to local context. However, WHO guidelines are often not successfully implemented in countries because of weaknesses and lack of coordination in country expertise and stewardship often resulting in competing efforts, fewer synergies, and low sustainability [18]. To address this challenge, WHO offices in the focus countries received technical support and catalytic funds to support FP programmes in countries. These funds leveraged additional resources from partners and ministries of health to strengthen the national FP programmes by updating/developing national norms and guidelines, training health care providers on FP/PPFP or task sharing (based on country needs) Fig. 2.

Early results show that the capacity building and the financial support provided to the country office acted as a catalyst to accelerate the development and implementation of national FP guidelines and tools. During the year 2016, all 10 focus countries revised/updated their national Family planning guidelines, tools, based on the latest WHO FP guidelines. In Kenya, Rwanda, Tanzania, Uganda, and Zambia 2015 MEC wheel was adapted to make a country specific wheel, while another 5 countries- Democratic Republic of Congo, Burkina Faso, Ethiopia, Madagascar, and Nigeria adopted the generic wheel as country job aid. Development of national guidelines and tools is the first step towards implementation of evidence based family planning service. WHO will continue to support implementation and monitor the use of these guidelines at national and subnational level and the impact that it may have on the life of women, children and adolescents globally.

Building WHO expertise also strengthened inter-country partnership. For example WHO Uganda country office facilitated and provided technical support to Zambia for the development of country standards for Family planning. This has been a major step forward in the implementation of the programme. Next year we will continue to support WHO country office, work with local partners in rolling out the guidelines and training at subnational level

## *Approach 4: Strengthening partnerships for introduction and implementation* of WHO guidelines

Partnerships can be effective vehicles for improving service delivery. Institutions within and outside public sector are important to sustain leadership, enable resilience to shocks and further the achievement of national development goals [19]. This project is designed to mobilize and engage commitments of domestic partners and networks like academia/trainers with disparate skills, strengths, disciplines not only to avoid confusion, duplication and redundancy but also for coordination of efforts and sustainability.

In all the 10 focus countries the MoH has engaged with and is actively collaborating with a range of partners including academia, scientific, implementing partners, donors like FP 2020, Track 20, local IBP partners, UNFPA, WHO, Population Council and JHPIEGO to advocate and build consensus for implementation of evidence based FP guidance. Relevant partners participated in the workshops and based on their skills/comparative advantages they agreed to support national FP programmes and services. In this initial phase of implementation, it was noted that there were significant tangible and intangible benefits and initial estimates indicate that there was up to three times leverage on account of this targeted funding. Leveraging was in the form of local partner’s contributions both in cash and kind For example in Ethiopia, USAID collaborated with MoH in training health care providers on PPFP; in Nigeria and Zambia UNFPA supported printing of the national FP guidelines and job aids like MEC wheel. In Kenya, JHPIEGO sponsored a workshop for the adaptation of the MEC wheel. In Burkina Faso, Ouagadougou partnership supported training of health care workers. In all 10 focus countries Implementing Best Practices (IBP) partners contributed to disseminate lessons learnt via the Knowledge gateway.

Early results (intermediate outcomes) show that although coordination with the different partners takes time and require alignment of different visions; it helps to build synergy, avoid duplication of efforts and has the potential to have a sustained response beyond the project life time in improving FP programmes and services thus delivering the vision of the SDGs and FP2020. Further achievements and impact of our work will be reported in the next manuscript on this project.

### Monitoring the progress

Monitoring is an important means to hold stakeholders accountable for their commitments to the achievements of global and national goals. Lack of meaningful data and failure to use the available data on PPFP has been reported as one of the major drawbacks that prevents improving quality of family planning services in the postpartum period^4^.

A base line survey was conducted to collect data on the awareness, knowledge and use of WHO guidelines in the countries. Key indicators such as the number of countries that have updated their national guidelines based on WHO standards; number of personnel trained to support national programmes; documentation of best practices and lessons learned during adaptation will be collected to monitor the progress and evaluate the overall success of the project. Additional indicators such as, the unmet need for contraceptives, contraception demand satisfied policy amendments, evidence of increase government commitment (e.g. funding, resources) will be used to show impact. All efforts will be made to collect data from the existing health information management system. Data collected will be compared with base line, evaluated so that lessons learnt can be used for scaling up services. The results will be share in a follow up manuscript.

As part of continuous improvement plan we have used various strategies to ensure strong participation and communication amongst all team members to achieve the set objectives. Therefore information and communication strategies are employed to ensure that the progress is continuously tracked and team members receive feedback and support (continuous information loop) to enable timely corrective actions which will result in improved performance leading to achievement of national goals. These strategies include weekly updates by the project manager on project activities shared by emails, monthly telephone calls to with focal points to receive regular project updates and assess technical support needs, webinars on technical updates and a newsletter published quarterly that helps to share experiences and address implementation challenges.

## Discussions

There is a paucity of rigorously evaluated processes to support countries in implementing WHO recommendations. Results from a recent survey expressed high level of reliance of countries on WHO’s technical support, either through consultants or staff [20] . WHO country focal points are often called to lead the development of national FP norms and standards; they oversee multiple programmes and often may not have the updates on the latest evidence. Thus building a critical mass of experts at country level is essential to ensure sustainability of programmes as confirmed by other programmes too [21].Feedback from WHO country offices at the end of the year reflects improved confidence, engagement and enhanced skills in SRH following the capacity building approaches. The implementation of the project started in early 2016. Even though, at this stage we cannot claim on the impact of these approaches in reducing contraceptive services; however some lessons can be distilled from the intermediate outcomes. These include the following:

*Leadership by Ministry of health is critical for ownership and sustainability*- Ensuring MoH in the driving seat is an efficient strategy to ensure commitment, harmonization and effective implementation in the countries. In the ten focus countries, greater commitment of government and implementing partners to evidence based family planning policies was observed. Agreement to key milestones, roles & responsibilities, active monitoring (tracking calendar of event, telephone calls, newsletter, and monthly meetings), effective communication and accountability at all levels helps in executing the planned activities on time. Going forward we will continue to support MoH and strengthen local partnerships particularly with USAID, UNFPA and FP2020.

*Skilled professional at country level is mandatory for sustainability of efforts*- A Regional focus to build a critical mass of skilled professionals/program managers at regional/country level to support MoH to adopt and implement the WHO guidelines is important. Various models can be used to build regional capacity. We employed regional workshops to strengthen skills of WHO country office, ministries of health and implementing partners. Country teams came together to discuss key challenges and made plans to improve the situation; country workshops led by national obstetrics and gynaecology society (FIGO-WHO Collaboration) leading to experts at country level to support ministries of health at national and subnational level; horizontal share of skills -country-country support for adaptation and development of national guidelines.

*Role of Catalytic funding in galvanizing efforts*- Financial support to WHO country offices (as seed money) led to increase visibility and engagement of WHO offices with partners and ministry of health. This resulted in ownership and accountability as well as leveraged additional resources and commitment from partners to support contraceptive and FP services in countries.

*Collaboration and coordination among implementing* partners is crucial for efficiency and effectiveness of efforts. Creating partnerships with all key stakeholders through consensus building led to ownership of the process and new and direct links for more effective implementation. Partners made significant commitment to the new guidelines and job aids developed in countries, agreed to use the tools developed for sub national implementation which is an important change from the past and will enhance actual resources available by avoiding duplication of activities.

## Limitations

At this early stage of project implementation, the outcomes (success factors) are mostly quantitative; number of national guidelines developed or capacity of regional experts developed in countries. It therefore is difficult to show results as outcome or impact at this stage. Based on the outcomes it is our expectation that the multiple approaches described above have the potential to strengthen country’s health systems and will contribute significantly to the identified targets of universal access to sexual and reproductive health.

## Conclusion

In summary, this project presented a unique opportunity for WHO, ministries of health and partners to actively collaborate to reorient and strengthen national family planning programmes and services. The gains made in this short time (development of national guidelines and building experts at country level) can be stated as intermediate success factors. The lessons learned reflect that simultaneous application of these approaches has the potential to create stronger partnerships for SRHR beyond the project life time and in delivering the vision of the SDGs and FP2020. The lessons learned may be used to scale up and expand contraceptive services in other countries.

## Abbreviations

DMT: Decision making tool for Family planning Clients
FIGO: International Federation of Gynecology and Obstetrics
FP: Family planning
IBP: Implementing Best Practices
MEC: Medical Eligibility Criteria for contraceptive use
MOH: Ministry of health
PPFP: Post-partum family planning
SDG: Sustainable Development Goals
SPP: Strategic Partnership Programme
SPR: Selected Practice Recommendations for contraceptive use
SRH: Sexual and reproductive health
ToT: Training of Trainers
UN: United National
UNFPA: United Nations Population Fund
USAID: United States Agency for International Development
WHO: World Health Organization

## Footnotes

1 Burkina Faso, Democratic Republic of Congo, Ethiopia, Kenya, Nigeria, Madagascar, Rwanda, Tanzania, Uganda, Zambia
