# Correction to: Design and initial implementation of the WHO FP umbrella project- to strengthen contraceptive services in the sub Saharan Africa

**DOI:** 10.1186/s12978-017-0406-2

**Published:** 2017-11-06

**Authors:** Rita Kabra, Moazzam Ali, James Kiarie

**Affiliations:** 1 Department of Reproductive Health and Research including UNDP/UNFPA/UNICEF/WHO/World Bank Special Programme of Research, Development and Research Training in Human Reproduction, World Health Organisation, Avenue Appia 20, 1211 Geneva, Switzerland; 2 Department of Reproductive Health and Research, World Health Organisation, Geneva, Switzerland; 3 22 chemin Henry Wissner, Grand Lancy, 1212 Geneva, Switzerland

## Correction

After publication of the original article [1], it came to the authors’ attention that the Acknowledgements section was not completed correctly. The Acknowledgements of the article should have been as follows.
